# Anxiety in Williams Syndrome: The Role of Social Behaviour, Executive Functions and Change Over Time

**DOI:** 10.1007/s10803-017-3357-0

**Published:** 2017-11-09

**Authors:** Elise Ng-Cordell, Mary Hanley, Alyssa Kelly, Deborah M. Riby

**Affiliations:** 1 Department of Psychology, Durham University, Durham, UK; 2 Psychology Department, Science Laboratories, Durham University, South Road, Durham, DH1 3LE UK; 3 Present Address: Department of Experimental Psychology, University of Oxford, Oxford, UK; 4 Centre for Developmental Disorders, Durham University, Durham, UK

**Keywords:** Williams syndrome, Anxiety, Longitudinal, Social functioning, Executive function

## Abstract

Anxiety is a prevalent mental health issue for individuals with Williams syndrome (WS). Relatively little is known about the developmental course of anxiety, or how it links with core features of WS, namely social and executive functioning (EF). In this study, parent-reports of anxiety were compared across a 4-year period (N = 17), and links between anxiety, social and EF were explored from concurrent parent-reports (N = 26). Results indicated that high anxiety persisted over time, and anxiety was related to impairments in both social and executive functioning. Importantly, results indicated that impairments in EFs may drive the links between anxiety and social functioning. This timely investigation provides new insights into anxiety in WS and highlights potential areas for intervention.

## Introduction

Williams syndrome (WS) is a sporadically occurring, relatively rare, developmental disorder caused by a hemizygous deletion of approximately 25–28 genes on chromosome 7q11.23 (Hillier et al. 2003). It has a reported prevalence of 1:20,000 (Korenberg et al. 2003, but see also 1:7,500; Strømme et al. 2002), and affects males and females equally. WS is associated with a distinctive profile of medical, physical, cognitive and behavioural characteristics. For example, individuals with WS can have medical difficulties including heart problems (supravalvular aortic stenosis), hypercalcemia, musculoskeletal abnormalities, and distinctive facial morphology (Morris 2006).While there is considerable variability of intellectual functioning, most individuals with WS have mild to moderate cognitive impairments (Mervis et al. 2000). Behaviourally, individuals with WS tend to be very friendly and empathetic, and have often been described as hypersociable (Jones et al. 2000). In more recent years there has been a focus on the social atypicalities (Lough et al. 2015) and psychiatric comorbidities, such as anxiety (Dykens 2003). Anxiety is the most prevalent mental health concern, especially by adulthood (e.g. Stinton et al. 2012). However, despite this heightened prevalence, interventions targeting anxiety are lacking (e.g. Cherniske et al. 2004). As future intervention strategies require a comprehensive understanding of anxiety in WS, the present study focuses on the phenomenology, development, and correlates of this phenomenon. It is important to consider how anxiety might be associated with the other characteristics of the disorder, specifically aspects of the combined cognitive and social phenotypes.

### Anxiety in Williams Syndrome

Alongside a socially gregarious disposition, the high prevalence of anxiety-related psychopathology is a seemingly paradoxical feature of WS. In a comprehensive study of anxiety in WS, Leyfer et al. (2006) assessed the occurrence of co-morbid psychiatric disorders in 4–16 year-olds with WS (N = 119). Using the Anxiety Disorder Interview Schedule (Silverman and Albano 1996), a structured clinical interview for parents, they found that rates of generalised anxiety disorder (GAD; 12%) and specific phobia (SP; 54%) were significantly higher than observed in the general population and those with intellectual disability. Furthermore, rates of GAD were higher among older individuals (11–16 years) than would be expected based on rates in younger individuals (4–6 years), suggesting anxiety in WS increases with age. Elsewhere, Cherniske et al. (2004) assessed the psychiatric profiles of 20 adults with WS. Based on diagnostic assessments by licensed psychiatrists, 13 individuals were classified as having moderate or severe anxiety, while three were described as having milder, subclinical problems. Again, the most common anxiety disorders were SP and GAD. By extending the previous findings to an adult sample, this study further supported the claim that anxiety is a persistent phenomenon in WS.

The studies outlined above employed cross-sectional designs, preventing inferences about the development of anxiety over time. To date, there are two known longitudinal studies of anxiety in WS. First, Woodruff-Borden et al. (2010) used the Anxiety Disorder Interview Schedule to assess 4–13 year-old children with WS (N = 45; mean age 6.67 years) in a two-year longitudinal study. While 60% of their sample presented with at least one anxiety disorder on initial assessment, this figure had increased to over 80% by follow-up. Seventy-two per cent of those with an initial anxiety disorder had developed an additional diagnosis at follow-up. Thus, the frequency of additional cumulative diagnoses suggests that in WS, anxiety generally remains stable, and in some individuals increases over time.

Not all findings support this trend though, as Green et al. (2012) conducted a five-year longitudinal study, exploring rates of psychiatric disorders among 6–23 year-old children with WS (N = 38) and developmental disabilities of mixed etiology. Using the Kiddie Schedule for Affective Disorders and Schizophrenia (Kaufman et al. 1997), they found that rates of anxiety disorders were significantly higher in WS at both time points. However, the WS group also showed a dramatic decrease in prevalence rates from initial assessment (84%) to follow-up (44%). A small proportion of the WS children in this sample received SSRI medication over the study period, which may have influenced outcomes. Furthermore, the average age of the sample at initial assessment was 13 years, as opposed to 6 years in Woodruff-Borden et al. (2010) study, and it may be that the trajectory of anxiety changes from childhood to adolescence. Given these differing accounts, further longitudinal studies across a wider age range are warranted to clarify the developmental trajectory of anxiety in WS.

### Anxiety and Social Functioning in Williams Syndrome

A defining feature of WS is the social profile, characterized by heightened attraction to faces and a significant motivation towards social interactions (Jones et al. 2000; Frigerio et al. 2006). Numerous reports describe people with WS as “hypersocial” compared to those with other developmental disorders and typically developing individuals (see Järvinen-Pasley et al. 2008). However, despite strong affiliative tendencies, individuals with WS consistently score within the range of mild-to-moderate impairment on social reciprocity measures (e.g. Kirk et al. 2013; Lough et al. 2015). These difficulties have lasting impacts on adaptive functioning and well-being. For example, undiscerning social approach behaviours—such as indiscriminately engaging others without considering social cues—coupled with cognitive impairments can increase the potential for victimization and social vulnerability (Klein-Tasman et al. 2011; Lough et al. 2015; Jawaid et al. 2012; Riby et al. 2017). Furthermore, many adults with WS are unable to maintain friendships, and suffer from social isolation (Howlin and Udwin 2006).

Attempts to understand the socio-emotional and behavioural correlates of anxiety in WS have led to an emerging body of research into the potential interplay between anxiety and social functioning. Riby et al. (2014) measured parental reports of anxiety using the Spence Children’s Anxiety Scale (SCAS; Spence 1998)—and social functioning—using the Social Responsiveness Scale (SRS; Constantino and Gruber 2005) in their study of 59 individuals with WS aged between 6 and 36 years. They found a small positive but significant correlation between anxiety and social functioning impairments (r = .362, *p* < .01). Splitting the participants into high- and low- anxious groups revealed that highly anxious individuals had greater impairments on the SRS subscales social awareness, cognition, and communication, which are said to reflect “socio-cognitive” functions (Klein-Tasman et al. 2011). Interestingly, social motivation did not differ between the groups. In other words, although both groups were similarly motivated by social interactions, the high-anxious individuals were less adept in socio-cognitive domains. This does not support the idea that social motivation serves as a protective factor against anxiety but rather that hypersociability may mask anxiety in social situations (Dodd et al. 2009; Dykens 2003). Therefore, when trying to understand anxiety in WS it is important to account for the role of social functioning.

### Anxiety and Executive Functioning in Williams Syndrome

Executive functions (EFs) have become a topic of increased focus within the WS cognitive profile. They are a group of higher-order cognitive processes associated with pre-frontal circuits believed to modulate cognitive, social, and emotional behaviours. They are widely conceptualized as a set of separate but related constructs, including cognitive flexibility (or shifting), inhibition, and working memory (Miyake et al. 2000). Several studies have found delays and impairments across a range of EFs in WS; including inhibition, set-shifting, and working memory (e.g. Menghini et al. 2010; Rhodes et al. 2010). Furthermore, while some developmental improvements in EF are observed during early childhood, deficits generally persist into adulthood (Greer et al. 2013).

Evidence from typical development indicates that executive dysfunction is associated with higher trait anxiety (Ursache and Raver 2014), and moderates the relationship between having an anxious temperament and developing an anxiety disorder (Fox 2010). In a study of adults with WS (N = 19) Rhodes et al. (2010) administered a battery of tasks measuring attention set-shifting, planning, and working memory abilities. They found that executive dysfunction across these tasks was associated with parental reports of negative affect, conduct problems, and decreased prosocial behaviours, as measured by the Conners Parent Rating Scale (Conners et al. 1998), and the Strengths and Difficulties Questionnaire (Goodman 2001).

Elsewhere, McGrath et al. (2016) explored the relationship between anxiety and attentional control in individuals with WS (aged 12–56), using the SCAS and a social dot-probe task in which participants (N = 46) were exposed to either happy or angry faces. They reported that highly anxious individuals displayed an increased bias towards angry faces, which was primarily explained by an inability to *disengage*, or shift attention from threatening stimuli. They noted that as general “sticky attention” effects are well-documented in WS (e.g. Riby and Hancock 2008; Riby et al. 2011).It is possible that a broader difficulty in attention-shifting, coupled with hypervigilance to threat, underlies the onset and maintenance of anxiety in this population.

In response to suggestions that performance-based EF tasks are too reductionist (e.g. Brown 2006), rating scales such as the behaviour rating inventory of executive functioning (BRIEF; Gioia et al. 2000) have been designed to measure EFs with greater ecological validity, by allowing researchers to assess regulatory abilities in everyday settings (Kenworthy et al. 2008). So far, the BRIEF has yielded several important findings in WS research. For example, Woodruff-Borden et al. (2010) found that the presence of an anxiety disorder was associated with increased behavioural and emotional dysregulation. Moreover, this relationship was stable over time. As the authors only reported difficulties in broader domains of behavior and emotion regulation, it would be interesting to build on these findings, honing in on the specific functions associated with increased anxiety.

More recently, Pitts et al. (2016) investigated the association between Specific Phobia (SP—as measured by the ADIS-P) and behavioural regulation (as measured by the BRIEF) in their sample of children and adolescents with WS (N = 194). Using a logistic regression model, they found that behavioural dysregulation was the strongest predictor of SP, with children at or above clinical levels of dysregulation at the greatest risk for SP. The authors proposed that impaired abilities to self-regulate or shift attention away from threatening stimuli leads to the subsequent development of irrational fears surrounding specific objects or situations. While this study has important implications for understanding some of the cognitive and behavioural foundations of anxiety in WS, its scope is limited as it only covers the associations between EF and SP. As such, it is unclear whether behavioural dysregulation confers an increased risk for both cue-specific and more generalized forms of anxiety (such as GAD). It is thus important to build upon this work by investigating whether the reported relationship extends beyond cue-specific anxiety disorders towards a broader range of anxious symptomatology.

Therefore, although the evidence suggests that EF impairments are implicated in the presence of anxiety in WS, further work is needed to address gaps in current understanding. For example, questions remain as to the relative contribution of different components of EF to the development and maintenance of anxiety in WS, and the associations to other key characteristics of WS, namely social functioning.

### The Current Study

In light of the above literature, the aims of this study were two-fold. The first aim was to explore the developmental course of anxiety in WS by looking at changes in its presentation over time (4 years) and its association with age (in the cross-sectional sample). Based on existing literature, anxiety was predicted to increase over time, and with age. A second aim was to explore how anxiety was associated with other core features of the disorder, namely social and executive functioning. It was predicted that most individuals with WS would show impairments in both social and executive functioning. Specifically, those with higher levels of anxiety were predicted to present with more difficulties with social functioning. We also explored whether specific aspects of executive functioning (i.e. shift, inhibit, etc.) were differentially associated with anxiety, although due to limited literature no specific predictions were made. Finally, the study sought to examine the extent to which social and executive functioning predicted anxiety in the cross-sectional sample. As no studies to date have measured all three aspects of the WS psychosocial profile concurrently, no specific hypotheses were posed.

## Method

### Participants

#### Cross-Sectional Sample

Participants were 26 parents or caregivers (25 mother, 1 older sibling) of individuals with WS who were aged 5–37 years (13 were female; 11 were under the age of 18). 21 individuals had received a genetic diagnosis via fluorescent in situ hybridisation testing and the remaining five had been diagnosed phenotypically on the basis of supravalvular aortic stenosis and facial dysmorphology before the availability of routine genetic testing. These 26 individuals are referred to as the “cross-sectional sample” for whom parental data were obtained.

#### Follow-up Sample

Within this sample, the parents of a subset of 17 individuals, aged 8–37 years (8 WS females) had participated in a previous study (“Time 1”; part but not all of the sample reported in Riby et al. 2014). This group of 17 is referred to as “the follow-up sample” for whom parental data were obtained. Data from the parents of these 17 individuals at Time 1 and at the current time point (“Time 2”) comprised the follow-up data set^1^.

### Measures

Data on anxiety, verbal ability (measured using the British Picture Vocabulary Scale II BPVS-II; Dunn et al. 1997) and non-verbal ability (measure using the Ravens coloured progressive matrices (RCPM); Raven et al. 1990) were available from Time 1. Anxiety, social functioning (measured by the Social Responsiveness Scale 2; Constantino & Gruber, 2012, and executive functioning (measured by the BRIEF; Gioia et al. 2015) were measured at Time 2 for all 26 participants.

#### Anxiety

The Spence Children’s Anxiety Scale—Parent Version (SCAS-P; Spence 1998) is a 38-item parent-report measure that assesses symptoms of anxiety based on DSM-IV criteria for childhood anxiety disorders (APA, 1994). It is reported to have good internal consistency, with an alpha coefficient of 0.92 (Spence et al. 2003). In the current sample, Cronbach’s alpha for the SCAS-P was 0.833. The items comprise six subscales–Panic/Agoraphobia, fears of physical injuries, separation anxiety, social phobia, obsessive–compulsive disorder, and generalized anxiety disorder. Parents rate each item on a four-point Likert Scale (*never, sometimes, often*, and *always*). Items are summed to produce an overall anxiety score, where higher scores indicate greater severity. While there are no standardized clinical cut-offs, a total score of 24 is one standard deviation above the mean in a community sample (Nauta et al. 2004). This was used as a cut-off point for clinical significance in previous WS studies (Rodgers et al. 2012; Riby et al. 2014). For parents of individuals over 18, an adult version of the SCAS-P was used in which some items were adapted by wording or content to be developmentally appropriate (for example, “other kids” was replaced with “other people”). The adult version of the SCAS-P retained the same structure as the original SCAS-P, as the total number of questions, and number of questions in each subscale, was the same across both measures. This exact adaptation of the items for adults has previously been used in published research (see Dodd et al. 2009; Riby et al. 2014). It is not unusual for child measures used to capture anxiety to be used with adults with WS (see both Porter et al. 2009 and Dykens et al. 2005 for the use of other anxiety measures designed for children but used with WS adults).

#### Social Functioning

The Social Responsiveness Scale Second Edition (SRS-2; Constantino and Gruber 2012) is a 65-item parent-report measure, designed to assess impairments of social reciprocity characteristic of ASDs. Its reported internal consistency is high, with an alpha coefficient of 0.95 (Constantino and Gruber 2012). Cronbach’s alpha in the current sample for the SRS was 0.806. The items map onto five subscales: social awareness, social cognition, social communication, social motivation, and restricted interests and repetitive behaviours. Higher scores indicate greater impairments. The SRS-2 provides two subscales corresponding with the two symptom domains within ASDs: social communication and interaction (SCI) and restricted and repetitive behaviour (RRB). Total and subscale scores can be converted to T-scores, which fall into three ranges of functioning: normal, mild-to-moderately impaired, or severely impaired. As T scores can reduce the spread of scores especially at higher levels of impairment, they were used for the purpose of classification into ranges of functioning and SRS raw scores were used for all other analyses (in line with Riby et al. 2014). For parents of individuals under 18, the SRS-2 School-Age Form was used. For parents of those over 18, the SRS-2 Adult Form was administered. The total number of items, and number of items within each subscale is consistent across both forms, allowing for data for both adults and children to be combined Constantino & Gruber, 2012). These data are referred to collectively as SRS-2 data.

#### Executive Functioning

The Behaviour Rating Inventory of Executive Functioning Second Version, Parent Form (BRIEF-2; Gioia et al. 2015) is a 63-item questionnaire for parents of 5-18-year-olds that measures everyday EF behaviours. Internal consistency is high across all scales and indices (all alpha coefficients above 0.90; Gioa et al. 2015). Cronbach’s alpha in the current sample for the BRIEF-2 was 0.949. Items map onto ten clinical scales: inhibit, self-monitor, shift, emotional control, initiate, task completion, working memory, plan/organize, task-monitor, and organization of materials. The scales comprise three indices: the Behaviour Regulation Index, Emotion Regulation Index, and Cognitive Regulation Index. For parents of individuals over 18, The Behaviour Rating Inventory of Executive Functioning Adult Version, Informant Form (BRIEF-A; Roth et al. 2005) was administered, as it is reported to be a more valid measure of EFs in adults with WS (Hocking et al. 2015). The BRIEF-A contains the same clinical scales as the BRIEF-2. Its reported internal consistency is high, with alpha coefficients of 0.80–0.98 (Roth et al. 2005). Cronbach’s alpha for the BRIEF-A was 0.975. For the purposes of this research, the scales Inhibit, Self-Monitor, Shift, and Emotional Control were used, as they map onto the Behavior and Emotion Regulation Indices.^2^

#### Combining BRIEF data

To increase power in the analyses, data from the BRIEF-2 and BRIEF-A were combined, and are referred to collectively as BRIEF data. This was done after consulting the authors of the manual (Isquith, personal communication, 18th July 2016). For each clinical scale, raw scores can be converted to T-scores, where higher scores indicate higher levels of dysfunction. Additionally, T-scores can be classified into four ranges of function/dysfunction: T-scores up to 59 indicate a normal level of functioning, between 60 and 64 indicate mildly elevated levels of dysfunction, between 65 and 69 indicate potentially clinically elevated levels, and at or above 70 indicate clinically elevated levels. As the number of items relating to each subscale differs between the BRIEF-2 and BRIEF-A, T-scores were used (instead of raw scores) for both categorization and analysis.

### Procedure

The project received ethical approval from the local ethics committee. Participants were families with a child with Williams Syndrome in Ireland and Northern Ireland and were recruited through the Williams Syndrome Association of Ireland and the Williams Syndrome Foundation UK. Parents wishing to participate opted in voluntarily to the project by signing an informed consent form, and were given a questionnaire pack with a stamped addressed envelope for return. A debrief sheet was attached for parents to read after completing the questionnaires.

### Analytic Approach

In the follow-up sample, SCAS-P scores at Time 1 and Time 2 (total and subscale) were compared using paired-samples t-tests, and change over time in anxiety scores was correlated with age and intellectual ability.

Corrections for multiple comparisons were not applied due to low power caused by the relatively small sample size in this study: an alpha value of 0.05 is applied throughout the manuscript, whereby a p value greater than 0.05 indicates a non-significant results. Furthermore, effect sizes are used throughout the Results section to aid the interpretation of the analyses (effect size for r are as follows − 0.1 is small, 0.3 is medium, 0.5 is large according to Cohen 1988; effect size for d are as follows − 0.2 small, 0.5 medium, 0.8 large, again according to).

In the cross-sectional sample, SCAS-P scores were correlated with chronological age. Next, the associations between anxiety, social functioning, and executive functioning were examined. Correlations between SCAS-P total scores and (1) SRS-2 and (2) BRIEF scores were undertaken. Finally, a backwards multiple regression was performed, using the Enter method. SRS-2 total raw score and BRIEF T-scores were included as the predictors, and SCAS-P total score as the dependent variable.

## Results

### Does Anxiety Change over Time?

Data for the follow-up sample are summarized in Table 1. Mean age difference from Time 1 to Time 2 was 3.75 years (*SD* = 0.43). At time point 1 mean RCPM score was 17.56 out of a maximum possible score of 36, and mean BPVS raw score was 84.59 indicating an average verbal mental age of 5 years 7 months. Neither RCPM nor BPVS scores were significantly correlated to SCAS-P total score at Time 1 (BPVS: *r*(15) = 0.273, *p* = .289; RCPM: *r*(15) = 0.045, *p* = .869) or Time 2 (BPVS: *r*(15) = 0.179, *p* = .492; RCPM: *r*(15) = − 0.305, *p* = .251). However, medium effect sizes for the correlations between Time 1 anxiety and BPVS score and Time 2 anxiety and RCPM scores were found. This suggests that greater anxiety may be associated with greater verbal ability, but that poorer non-verbal ability is associated with greater anxiety at follow-up. This appears to present a conflicting picture in terms of the associations between cognitive abilities and anxiety based on effect sizes and is interpreted with caution.

**Table 1:** Demographic and anxiety data for the follow-up sample

Measure	Time Point						
	Time 1		Time 2		Difference (T2-T1)		
	Mean (SD)	Range	Mean (SD)	Range	*t*(16)	*p*	*d*
Demographics							
N	17	–	17	–	–	–	–
Age	19.12 (9.08)	4–34	22.86 (8.99)	8–37	–	–	–
Gender distribution	8 F, 9 M	–	8 F, 9 M	-–	–	–	–
BPVS (raw)	84.59 (24.58)	53–128	–	–	–	–	–
RCPM (raw)^a^	17.56 (4.91)	10–28	–	–	–	–	–
Anxiety SCAS-P raw scores							
Total	25.82 (12.07)	11–59	30.06 (11.59)	13–64	1.579	0.067	0.383
Panic/agoraphobia	3.41 (2.96)	0–11	3.76 (3.54)	0–14	0.485	0.634	0.109
Separation anxiety	4.65 (2.50)	2–11	5.71 (3.14)	1–14	1.450	0.166	0.376
Physical injury fears	5.71 (5.96)	1–26	4.47 (1.97)	1–9	−0.898	0.383	0.312
Social phobia	3.59 (3.45)	0–13	5.29 (3.77)	0–11	2.022	0.060	0.492
OCD	3.06 (2.41)	0–7	3.35 (2.96)	0–11	0.389	0.702	0.110
GAD	6.59 (2.85)	3–14	7.47 (2.94)	5–16	1.268	0.223	0.305

^a^RCPM data were collected for 16 individuals at Time 1

**Fig. 1:**
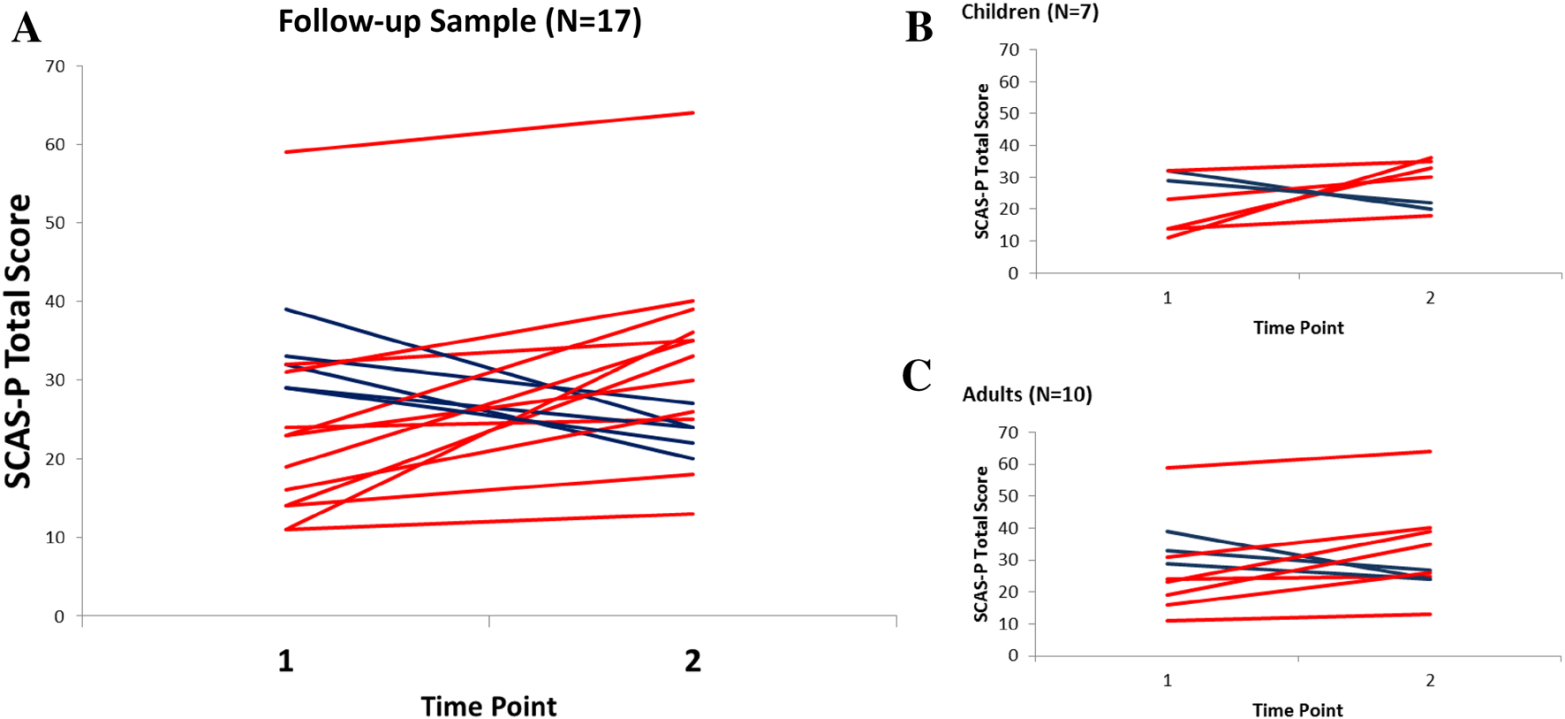
Total scores from the SCAS presented for each individual from the longitudinal sample for Time 1 and Time 2 (**a**), and separately for children under 18 years (**b**) and adults over 18 years (**c**), indicating change over time at the individual level. Lines in red show an increase and lines in blue show a decrease in anxiety. (Color figure online)

The mean total SCAS-P scores at Time 1 and Time 2 for the group were above the suggested cut-off for clinical elevation of 24, increasing on average by 4.24 points over the four year period. Paired-samples t-tests indicated that this increase approached significance, with a small effect size, however, there was significant individual variability (see Table 1). Twelve individuals (71% of the follow-up sample) increased in total anxiety from Time 1 to Time 2; five (29%) decreased in total anxiety. Among those who became more anxious, six individuals’ total scores increased by 9 points or more, which is 1 SD based on a normative sample (Nauta et al. 2004). Among those who became less anxious, two individuals’ total scores decreased by nine points or more (see Fig. 1 for individual changes).

The change in anxiety (calculated as the difference between total SCAS-P scores at Time 1 and Time 2) varied widely across the sample, ranging from a decrease of 15 points to an increase of 25 points. This change was not significantly correlated with chronological age, *r*(15) = − 0.076, *p* = .771, BPVS scores, *r*(15) = − 0.110, *p* = .674, or RCPM scores, *r*(15) = − 0.356, *p* = .177. Again, it should be noted that the effect sizes for these correlations point towards a possible association between a greater increase in anxiety and lower non-verbal ability.

**Fig. 2:**
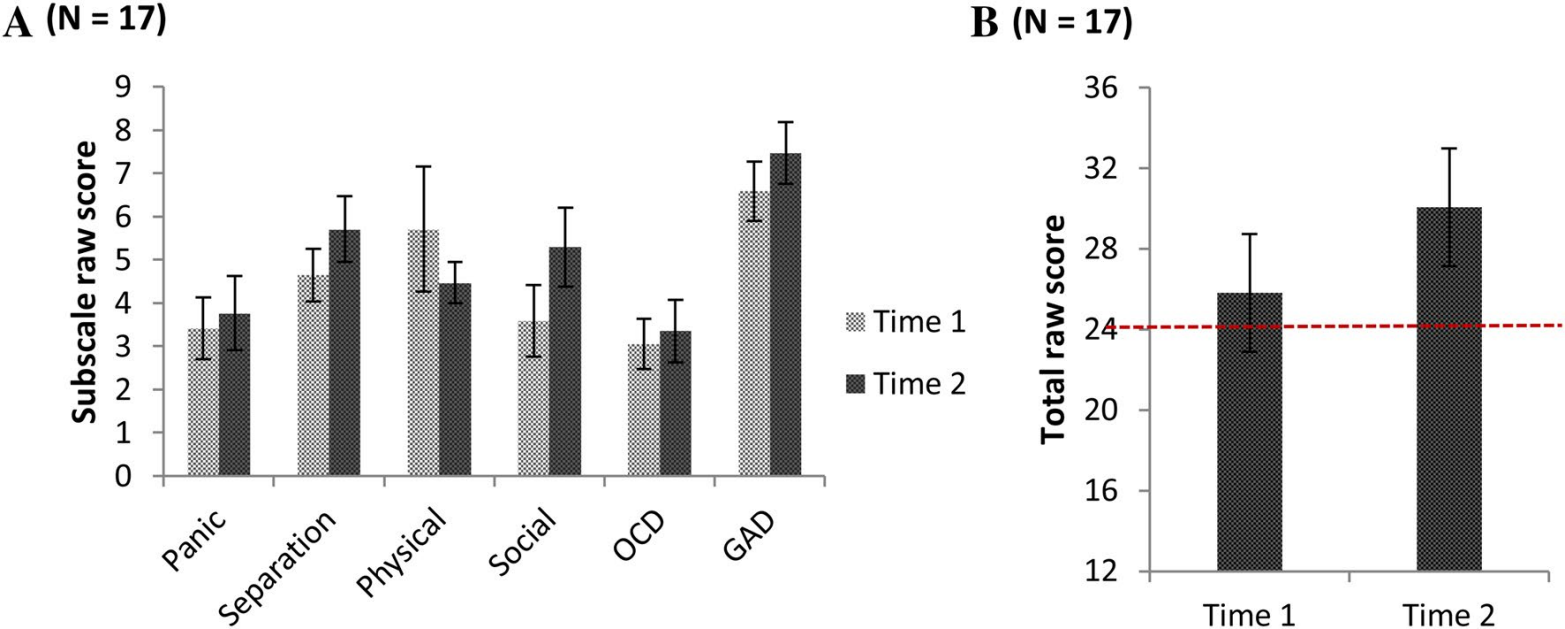
Scores on the SCAS_P subscales (**a**) and total scores (** b**) for time 1 and time 2, with the red line in ** b** indicating the cut-off for clinically high levels of anxiety

Changes in SCAS-P subscale scores for the group are displayed in Fig. 2. Scores increased from Time 1 to Time 2 across all subscales, with the exception of Fears of Physical Injury. Paired-samples t-tests (see Table 1) indicated that the changes were not statistically significant for Panic Disorder, Separation Anxiety, Physical Injury, OCD, and GAD. There was a trend towards significance for the change in Social Phobia. Effect sizes indicate there are likely to be clinically relevant increases in Separation Anxiety, OCD, GAD, and Social Phobia over time.

### Profile of Anxiety in the Time 2 Sample

Data for the cross-sectional sample are summarized in Table 2. Mean SCAS-P total score was 7.58 points above the suggested cut-off for clinical elevation of 24. Nineteen individuals (73% of the sample) scored at or above the cut-off, and were classified as “high-anxious”, while seven (27%) scored below 24, and were classified as “low-anxious”. Anxiety scores did not differ between (M = 30.23, SD = 13.39) and females (M = 32.92, SD = 14.25), *t*(24) = 0.496, *p* = .624, *d* = 0.195. The correlation between total anxiety score and chronological age was not significant, *r*(24) = 0.202, *p* = .161, nor were the correlations between SCAS-P subscale scores and chronological age (Panic Disorder: *r*(24) = 0.041, *p* = .843; Separation Anxiety: *r*(24) = 0.084, *p* = .683; Physical Injury: *r*(24) = 0.011, *p* = .957; Social Phobia: *r*(24) = 0.370, *p* = .063; OCD: *r*(24) = 0.315, *p* = .117; GAD: *r*(24) = 0.028, *p* = .892). There was a trend towards significance for the correlation between Social Phobia and age. Moreover, medium effect sizes for the correlation between age and Social Phobia, as well as OCD and total anxiety score suggest that overall anxiety along with OCD and Social Phobia may increase with chronological age in the sample.

**Table 2:** Demographic, SCAS-P, SRS-2, and BRIEF data for the cross-sectional sample

Measure	Mean (SD)	Range
Demographics		
N	26	–
Age	19.57 (9.66)	5–36
Gender distribution	13 F, 13 M	–
Anxiety SCAS-P raw scores		
Total	31.58 (13.62)	7–64
Panic/agoraphobia	4.65 (4.52)	0–20
Separation anxiety	5.73 (3.26)	1–14
Physical injury fear	4.96 (2.41)	1–12
Social phobia	5.08 (3.74)	0–14
OCD	3.65 (3.25)	0–12
GAD	7.88 (3.19)	4–16
Social functioning SRS-2 raw scores		
Total	83.96 (23.56)	34–125
Social awareness	9.73 (2.86)	3–17
Social cognition	19.04 (5.79)	5–29
Social communication	26.00 (8.58)	8–46
Social motivation	10.04 (4.70)	3–21
Autistic mannerisms	19.15 (6.28)	5–30
Executive functioning BRIEF T-scores		
Inhibit	65.38 (14.34)	40–89
Self-Monitor	66.15 (10.90)	44–84
Shift	69.27 (12.17)	52–90
Emotional Control	68.12 (13.01)	41–84

### How is Anxiety Related to Core Features of WS?

#### Anxiety and Social Functioning

On the SRS-2, five individuals (19% of the cross-sectional sample) were reported to show normal levels of overall functioning, 16 (62%) were reported to have mild-to-moderate impairments, and five (19%) were reported to have severe impairments. Chronological age was not significantly correlated with SRS-2 total score, *r*(24) = − .022, *p* = .916, suggesting that social abilities were not associated with chronological age in WS. Pearson’s correlations were calculated between SCAS-P total, and SRS-2 total. There was a significant positive correlation found between SCAS-P total and SRS-total with a medium effect size, *r*(24) = 0.353, *p* = .038, indicating that greater atypicality of social function is associated with higher anxiety.

#### Anxiety and Executive Functioning

Across all four BRIEF scales, the modal category of function/dysfunction was clinically elevated; 35–50% of the sample fell into this range, while 23–35% fell into the normal range. Chronological age was not significantly associated with T-scores on any of the BRIEF clinical scales (Inhibit: *r*(24) = − 0.307, *p* = .127; self-monitor: *r*(24) = 0.005, *p* = .980; shift: *r*(24) = 0.162, *p* = .429; emotion regulation: *r*(24) = − 0.174, *p* = .395), However, interpretations based on the effect sizes of these correlations indicate better Inhibition was associated with increasing chronological age.

There was a significant positive correlation between total anxiety score and T-scores for Inhibit, *r*(24) = 0.449, *p* = .011, Shift, *r*(24) = 0.625, *p* < .001, and emotional control, *r*(24) = 0.407, *p* = .019, with medium to large effect sizes. The correlation between total anxiety and Self-Monitor was not significant, *r*(24) = 0.124, *p* = .273.

#### Social and Executive Functioning

Exploratory Pearson’s correlations were performed to investigate potential relationships between SRS-2 total scores and each of the four BRIEF scales. Significant positive correlations with medium to large effects sizes were found between SRS-2 total and all four scales on the BRIEF, Inhibit, *r*(24) = 0.611, *p* < .001, Self-Monitor, *r*(24) = 0.642, *p* < .001, Shift, *r*(24) = 0.725, *p* < .001, and Emotional Control, *r*(24) = 0.550, *p* = .002. suggesting strong associations between social and executive functioning in WS. These correlations held when only the Social Communicative Index (SCI) from the SRS was used, therefore indicating that the associations were not driven by repetitive behaviours [SCI & Inhibit, *r*(24) = 0.613, *p* < .001, SCI & self-monitor, *r*(24) = 0.644, *p* < .001, SCI & shift, *r*(24) = 0.689, *p* < .001, and SCI & emotional control, *r*(24) = 0.497, *p* = .005].

#### Predicting Anxiety from Social and Executive Functioning

In the regression analysis, the following variables were included as predictors, as they were significantly correlated with the dependent variable (SCAS-P total): SRS-2 total, and BRIEF- Inhibit, Shift, and Emotional Control (*see* Table 3). The initial model generated (adjusted *R* ^2^ = 0.311) was a significant predictor of overall anxiety score, *F*(4,21) = 3.823, *p* = .017. Once the remaining variables were controlled for, SRS-2 total (*p* = .323), Inhibit (*p* = .649), and Emotional Control (*p* = .793) were not significantly related to total SCAS-P score (partial correlations: *r*s < 0.100, *p*s > 0.323). In other words, the unique variance between each of these predictors and total anxiety score was non-significant. However, Shift was found to be a significant predictor of SCAS-P total score, β = .702, *t*(21) = 2.595, *p* = .017. The positive Beta weighting for this coefficient indicated that those with greater problems in this domain had higher anxiety levels (Table 4).

**Table 3:** Predicting anxiety from social functioning and executive functioning – initial model

Predictor Variables	Outcome measure					
	Overall Anxiety^a^ (N = 26)					
	β	*t*	Partial Correlation	*R* ^*2*^ change	Adjusted *R* ^*2*^	*F* (4,21)
Model summary				0.421	0.311	3.823*
SRS-2 total raw score^b^	− 0.255	− 1.012	− 0.216			
Inhibit T-score^c^	0.109	0.461	0.100			
Shift T-score^c^	0.702	2.595*	0.493			
Emotional Control T-score^c^	0.059	0.266	0.058			

*p < .05

^a^Spence Children’s Anxiety Scale-parent form

^b^Social Responsiveness Scale-second version

^c^Behaviour rating inventory of executive function

**Table 4:** Predicting anxiety from social functioning and executive functioning – final model

Predictor variables	Outcome measure					
	Overall anxiety^a^ (N = 26)					
	β	*t*	Correlation	*R* ^*2*^ Change	Adjusted *R* ^*2*^	*F* (1,24)
Model summary				0.390	0.365	15.345**
Shift^b^	0.625	3.917**	0.625			

**p < .01

^a^Spence Children’s Anxiety Scale-parent form

^b^Behaviour rating inventory of executive function

## Discussion

This study aimed to investigate changes in anxiety in WS over a 4 year period, and to explore how anxiety was associated with other core features of the disorder, namely social and executive functioning. As predicted, and in support of the existing literature (e.g. Leyfer et al. 2006; Woodruff-Borden et al. 2010), over 70% of the current cross-sectional sample scored above the specified cut-off for clinically elevated anxiety levels. This was higher than the 42% reported by Riby et al. (2014), and the 38% reported by McGrath et al. (2016), although a recent meta-review by Royston et al. (2016) show that prevalence rates for anxiety disorder in WS have been reported to be as high as 82.2%. Therefore, our results further emphasise that anxiety is a significant issue for many individuals with WS. Additionally, our results indicated that most individuals became more anxious over time. The overall change in anxiety was not significant, although there was a trend towards significance, with a small to medium effect size. Effect sizes also indicated increases in several aspects of anxiety, such as separation anxiety, OCD, GAD, and SP. From this we can provide tentative support for the idea that anxiety is a chronic issue that worsens over time, but future studies with larger samples are warranted to more fully understand the developmental trajectory (Woodruff-Borden et al. 2010). Additionally, there was considerable individual variability in how anxiety changed over time in the follow-up sample, and a small proportion (one-third) of the sample became less anxious over time. This highlights a need to examine systematically potential risk and protective factors driving these longitudinal changes.

One aim within this study was to explore the role of age in anxiety, as reports in the literature are mixed as to whether anxiety increases with age. We did not find a clear correlation with age, and therefore propose two alternative explanations. On the one hand, given the relatively small sample size, the lack of significance may have been due to insufficient power. We note that the effect sizes for the correlations between age and the change in anxiety over time/anxiety in the cross-sectional sample at time 2 were both small (≤ 0.2). However, the effect sizes for the association between age and some of the anxiety subscale measures were medium to large, highlighting that there may be age-related changes in aspects of anxiety such as social phobia and OCD. Alternatively, sample differences could underlie the conflict with existing reports of age-related increases in anxiety (e.g. Leyfer et al. 2006). Leyfer et al. (2006) only included participants up to age 16, and therefore could not extend their findings to adults with WS. In contrast, the current sample had a much wider age-range. Indeed, lack of association between anxiety and age has also been reported in similarly composed samples (Riby et al. 2014), and it is possible that a quadratic relationship exists between anxiety and chronological age, such that anxiety increases from childhood to adolescence, and subsequently decreases from adolescence into adulthood. Future studies with larger samples could bring clarity to this issue by comparing the presentation of anxiety in WS between different age groups, and by paying particular interest to aspects of anxiety such as social phobia and OCD.

### Anxiety and Social Functioning

Our results showed that higher anxiety was associated with greater impairments in overall social functioning, in line with previous reports (Kirk et al. 2013; Riby et al. 2014). It has been suggested that difficulties in social functioning in ASD contribute towards anxiety via increasing difficulties in navigating social situations and subsequent social isolation (White et al. 2009). This may also be the case in WS, particularly if we take into account the characteristic strong desire for social interaction (Ng et al. 2014a). Given these results, incorporating social-skills training into the design of anxiety interventions in WS, as has been done in ASD (e.g. Wood et al. 2009), is worth consideration. As these findings are correlational, further prospective work is required to delineate the directions and trajectories of these relationships.

### Anxiety and Executive Functioning

Corroborating and extending the existing literature (e.g. Woodruff-Borden et al. 2010), greater impairments in EFs—specifically inhibit, shift, and emotional control—were associated with higher levels of anxiety. In light of these findings, it is important to consider the role these specific functions play. For example, poor inhibitory control is postulated to underlie anxiety symptoms such as repetitive questioning (Green et al. 2012), while poor emotional control may underlie emotional outbursts during distressing situations (Ng et al. 2014b). If so, then dysregulation across these domains might explain increased anxious symptomatology. Future studies would be required to substantiate these findings and prospectively study the direction of these associations. Nonetheless, these results raise the possibility of targeting anxiety in WS with a range of EF-based interventions, which have received growing empirical support for improving cognitive, social, and emotional outcomes in typically developing children (see Diamond and Lee 2011).

### Predicting Anxiety from Social and Executive Functioning

A unique aspect of this study was the integration of social and executive functioning in a model predicting anxiety in WS. Regression analysis revealed that when controlling for the other variables, Shift alone remained a significant predictor, reflecting a strong independent relationship between anxiety and cognitive flexibility. A crucial next step is to consider the pathways by which this ability relates to anxiety. According to attentional control (AC) theory (Derryberry and Reed 2002), excessive anxiety upsets the balance between stimulus-driven and goal-driven attention processes, causing over-responsiveness to, and biased processing of threatening stimuli. More recently, researchers have focused on subtypes of AC mechanisms, demonstrating that the ability to *shift* rather than *focus* attention is related to biased processing of threatening stimuli in anxiety (Taylor et al. 2016).

It is possible that in WS early difficulties in attention-shifting underpin a cycle of hyperawareness to and fixation on threat, resulting in elevated anxiety (McGrath et al. 2016). However, an alternative account has been raised by Kirk et al. (2013), who used the SCAS and a facial expression task in an eye-tracking study exploring the relationship between anxiety and social attention in WS. They found that anxious individuals with WS were initially over-attentive to, but subsequently avoided threatening stimuli (angry faces) by allocating attention elsewhere, suggesting a dual process of vigilance and avoidance (see Mogg and Bradley 1998). These differing accounts warrant further investigations to shed light on the precise mechanisms by which attentional and executive processes influence anxiety in WS. These efforts may guide the application of existing attention-based treatments for anxiety, such as Attention Bias Modification (ABM; see Lowther and Newman 2014), a computerized programme which involves training one’s attention to avoid negative stimuli.

One particularly intriguing finding casts new light on the relationship between anxiety and social functioning in WS. The regression model revealed that once shared variance in EF abilities were controlled for, the positive association between anxiety and social functioning no longer held. This raises the possibility of a mediating effect of EF in the relationship between anxiety and social functioning in WS. Turning to the literature on typical development, early executive dysfunction (poor inhibitory control, high impulsivity, and poor adaptive and attentional flexibility) has been found to predict poor social competence *and* externalizing and internalizing problems later in life (see Eisenberg et al. 2000). Furthermore, associations between social behaviours and EF in WS have been investigated elsewhere. Most notably, evidence that that impaired inhibitory control underlies inappropriate social approach behaviours (Little et al. 2013) supports the frontal lobe hypothesis (Porter et al. 2007).

Indeed, exploratory correlations between the SRS-2 and the BRIEF revealed significant, positive relationships across all four scales, further supporting the notion that EF underlies both social and emotional outcomes in WS. We stress that these findings are only preliminary, and must therefore be interpreted cautiously and substantiated with further systematic research. Nonetheless, they provide a compelling rationale for future efforts to prospectively study the development of EFs in WS, with a focus on exploring potential cascading effects on a range of psychopathological outcomes. It would be particularly beneficial if social *and* emotional vulnerabilities could be identified and targeted for intervention, based on early indicators such as executive dysfunction.

### Considerations and Future Research

Several considerations should be addressed in future research. One challenge to working with individuals with WS is the rarity of the disorder. The relatively small sample size in the current study may have limited power to detect statistically significant effects. One way to overcome this in future work would be to engage in collaborative multisite studies. Larger scale work on this issue would allow analysis to probe the potential mediating effects of executive functioning on the association between social functioning and anxiety, and findings where effects sizes indicated further consideration on a larger scale is necessary (association with non-verbal ability and anxiety). Another issue pertains to our reliance on measures of anxiety designed for individuals without neurodevelopmental disorders (e.g. SCAS-P). Attempts to capture more sensitively the anxiety profile in WS could utilize new measures designed for individuals with ASDs, for whom anxiety is similarly characterized by issues with sensory hypersensitivity and worry in anticipation of upcoming events (e.g. Rodgers et al. 2016). Ideally, parallel scales designed for children with developmental disorders *and* adults would facilitate the meaningful measurement of anxiety across the wide age-range in this sample. However, such measures have yet to be developed and validated empirically. Therefore, in recognizing this limitation, we highlight the need to develop targeted assessment measures.

Additionally, reliance on parental insights on questionnaire measures may lead to retrospective or subjective biases (see Dykens 2003), while the use of a single respondent may result in shared variance across the three measures. One way to address these issues in future would be to collect data from multiple informants (e.g. Klein-Tasman et al. 2011), or use self-report measures (e.g. Freeman et al. 2010). However, this can be especially challenging for younger individuals and those with lower intellectual capabilities. Moreover, parent-report measures are often used in clinical assessments of atypically developing populations, and we believe that the parents in the current study were able to adequately report on their children’s behaviours and functioning. Finally, it is important to take into account the potential for self-selection on studies of anxiety: for example, parents of particularly anxious individuals, or those with greater concerns about their child’s anxiety problems, may have been more likely to respond. However, given the considerable individual variation in overall anxiety scores, we believe the current sample provides a sufficiently balanced portrayal of the WS anxiety profile.

In conclusion, the current study highlights that heightened anxiety persists for many individuals with WS over time, and that executive functions may play an important role in this. We have proposed a top-down influence of executive functions on both anxiety and social functioning, and recommend future prospective investigations to study the downstream effects of early neuropsychological functioning in WS. Knowledge about the presentation and development of anxiety in WS, as well as dynamic associations with cognition and behaviour are imperative for the development of appropriate prevention and intervention strategies.

## Footnotes

1 One individual had a comorbid diagnosis of ADHD. Removing this person from the sample did not affect the pattern of results; therefore the entire sample’s data were retained in the analyses.

2 Inhibit measures the ability to control impulses and stop certain behaviours at appropriate times. Self-Monitor measures the ability to monitor the effects of one’s behaviour on others. Shift assesses the ability to move fluidly from one situation to another, and to solve problems flexibly. Emotional Control measures the ability to modulate emotional responses appropriately.
